# Effects of duration of uninterrupted fast in weekly intermittent fasting: Comparison of an 82-week 5:2 case report to an isocaloric modified 4:3 protocol

**DOI:** 10.21203/rs.3.rs-3701752/v1

**Published:** 2023-12-07

**Authors:** Katarina Borer

**Affiliations:** University of Michigan–Ann Arbor

**Keywords:** intermittent fasting, appetite, uninterrupted fast, weight loss, fat loss, energy expenditure, sleep quality

## Abstract

Intermittent fasting (IF) approach for weight loss obviates the inconvenience of calorie counting of daily caloric restriction (DCR). It tests IF ability to better counteract a metabolic defense mechanism (MDM) than DCR. MDM obstructs weight loss and facilitates weight regain possibly by increasing hunger and efficiency of exercise energy expenditure (EEf), and by reducing resting metabolic rate (RMR) and physical activity (PA). A knowledge gap exists about whether the duration of weekly uninterrupted fasts (UFs), where the IF protocols are isocaloric, mitigate the MDM. This study compares two IF protocols that have the same weekly number of hours of fast (108) and free access to food (60), but which differ in the duration of UF. An 82-week case report was conducted with twice-weekly near-absolute 36-hour fasts on non-consecutive days (5:2-NC) and compared to ten studies with a 20-hour UF on three non-consecutive days (4:3-NC) modified through provision of a 500–600 kcal meal on fasting days. The large meal raised insulin concentration for 4 hours and reduced the UF to 8 hours followed by 12 nocturnal hours of fasting. The hypotheses were that (1) because of their matched F/E ratio, the rates of weight and fat losses will be similar in both protocols, and (2) because of its longer UF period, hunger will be higher and RMR and voluntary physical activity lower, in 5:8-NC than in M4:3-NC protocol,. The main differences between the two protocols were, (1) slower rates of weight and fat losses, (2) lower sensation of hunger and substantial decline in fullness, no change in RMR and physical activity, and 2.5 times higher post-fast concentration of the ketone body beta-hydroxybutyrate (BHB) in 8:2-NC compared to M4:3-NC protocol. Absence of increased hunger and the variability of the rate of weight loss in 5:2-NC protocol, plus increased EEf in one M4:3-NC study suggest that IF does not curtail MDM, but shortened UF period in M4:3-NC reduces elicitation of BHB. Thus, the addition of a large meal on fasting days is unnecessary for prevention of hunger and is counterproductive for increases in BHB and its potential health benefits.

## 1. Materials and methods for the IF 5:2 NC protocol

### Subject.

A single subject carried out a self-directed IF 5:2-NC protocol over 574-days or 82-weeks. The subject’ baseline characteristics are shown in Table 1. The only medication used daily was 12.5 mg of hydrochlorothiazide diuretic, and 2 mg of a non-prescription sleep aid melatonin before bedtime.

### Dietary intake.

A Mediterranean dietary pattern was followed on the non-fasting days. On four days, breakfast included baked oatmeal with apples and sour-cream, nuts (almonds, hazelnuts, walnuts), kefir, pancakes with berries, and cottage-cheese crepes sweetened with Stevia. On one day, it was a vegetable omelet. Beverage was coffee with milk or tea. Lunch was Swiss cheese or canned tuna fish on toast with avocado, onion, and tomato, or home-made bone or bean soups. Dinners included 2 cups of mixed salad with vinaigrette dressing, brown rice, a green vegetable, and chicken or fish (salmon, cod, tilapia). Apples, grapes, pineapple, mango, nuts, occasional dark chocolate, cookies, or home-baked pies were eaten for desert. The beverage was tea or coffee with milk. Meals were eaten at 7, 12, and 16:30 h. Total fasting energy allotment on Tuesdays and Fridays was 87 to 96 kcal. It consisted of a 110–112 g slice of cantaloupe (about 40 kcal) and 3 hazelnuts (9 kcal/1.25 g nut, 85% fat, each) divided into three meals. Breakfast beverage was 125 ml of low-sugar orange juice containing 5 g of sugar (20 kcal), while green tea or water were consumed at other times.

### Anthropometric measures.

Body weight was measured daily at 7 h on a physician’s mechanical beam scale (Ava Weigh MSB440 lb). Body fat, fat-free or lean body mass (LBM), were measured on a bioimpedance scale (Tanita Wb-100a) eight times, at the start, and on days 185, 257, 345, 412, 450. 505, and 574. Height was measured intermittently with a stadiometer in the health center. Plasma glucose, hemoglobin A1c, triglycerides and low-density and high-density cholesterol were measured by the university hospital lab during week 1 and week 82.

**Appetite assessments** utilized the 100-cm visual analog scale (VAS) method [31]. Meals were restricted to a 10-hour eating window between 7 and 17 h. Hunger and appetite were assessed daily between days 225 and 251 at the following times: 7, 9, 11, 13, 15, 17, and 19 h.

**Blood pressure** was measured once a month at 7 h with an Omron Evolv wireless upper-arm blood pressure monitor (www.omronhealthcare.com).

### Energy expenditure measures.

Physical activity, daily energy expenditure, and sleep quality were measured daily with a Fitbit Versa 2 activity tracker and its software (www.fitbit.com) accessible on the cell phone daily and from the Fitbit weekly email reports. The device tracked daily number of steps and distance travelled. Besides daily walking, the subject engaged in two hours per week of Ashtanga yoga and resistance exercise using weight machines in the university gym. RMR was measured with the Tanita bioimpedance apparatus on days 185, 257, 345, 412, 450. 505, and 574.

**Sleep quality** was assessed from daily records of minutes of sleep collected by Versa-2 fitbit activity monitor. The variables tallied in nightly minutes were total, light (stages N1 and N2), deep (stage N3), and rapid-eye-movement (REM) sleep, and of awake periods during the sleep period, as well as number of brief awakenings.

**Beta-hydroxybutyrate (BHB)** was measured in lanced blood samples applied to ketone strips with the Precision Xtra blood glucose and ketone monitoring system (Abbott Diabetes Care Inc, Alameda CA). Measurements were done on days 251 through 265 between 07:00 and 07:30 h after the 38-h fast day and on the three post-fast eating days, D1, D2, and D3. A single measurement was done on day 266 after 60 hours of UF resulting from two consecutive days of fast.

### Statistics.

All daily measurements were plotted, and a linear regression were performed in SAS (Statistical Analysis System, sas.com/en_us, version 9.4, SAS Institute, Cary, NC, USA), and with GraphPad Prism software (https://www.graphpad.com/scientific-software/prism), version 9.5.0 (730). Figures of linear variables were graphed with Excel and GraphPad Prism software, while non-linear variables were plotted with LOESS procedure (https://peltiertech.com/loess-smoothing-in-excel).

## 2. Results for the for the IF 5:2 NC protocol

### Body weight changes.

**Figure 1:**
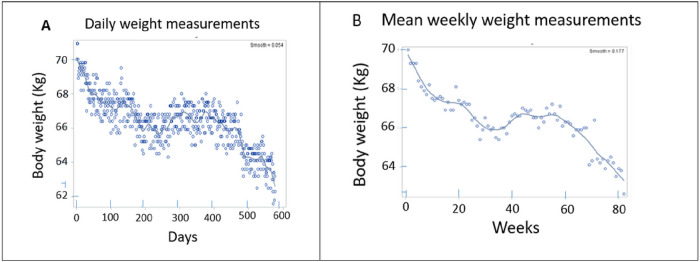
Daily (A) and mean weekly (B) weight changes.

Daily body weight changes are shown in Fig. 1A, and mean weekly changes in Fig. 1B. Weight loss was sustained through the 82 weeks of IF, and, by ANOVA, the time parameter of days of weight change shown in Fig. 1A was significant (F_(df=559)_ = 776.4, p < 0.0001; t=−27.9, p < 0.0001, and R^2^ = 0.5806. Similarly, the weekly weight change parameter was also significant (F_(df=559)_ = 80, p < 0.0001; t=−13, p < 0.0001, and R^2^ = 0.6747). Total weight lost in 82 weeks was 7.7 kg (0.09 kg/week) or 11.0% of starting weight. The LOESS plot of weekly weight changes in Fig. 1B shows that the rate of weight loss during 82 weeks was variable. There were three periods of rapid loss, the first 10 weeks (−0.25 kg/w), weeks 20 to 30 (−0.09 kg/w), and weeks 58 to 80 (−0.19 kg/w). In between, the rate of weight loss was lower between weeks 10 and 20 (−0.01 kg/w), unchanged between weeks 30 and 37 and 44 and 58, and even increased between weeks 38 and 44 (+ 0.25 kg/w).

### Changes in body fat mass, lean body mass, and BMI.

**Figure 2:**
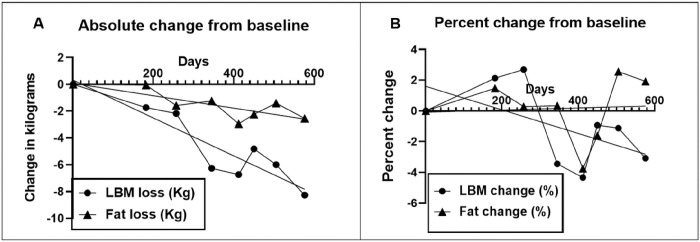
Absolute changes in fat mass and lean body mass (Kg) (A) and relative changes normalized by body mass (B).

Absolute changes in body fat and LBM are shown in Fig. 2A and changes normalized by body mass in Fig. 2B.

Total fat mass lost in 82 weeks was 2.55 kg or 0.03 kg/w. Linear regression (y=−0.004641*x + 0.08149) for the absolute change in body fat mass (Fig. 2A) was significant (F_(df=1.6)_ = 11.16, p < 0.0156). However, there was no change (y = 0.0006954*x-0.08714) in the body fat mass normalized to body mass (F_(df=1.6)_ = 0.0248, p = 0.88) (Fig. 2B). ). Percent fat mass lost over 82 weeks was 2.8 or 0.03%/w.

Total LBM loss was 8.3 ± 0.1 kg and its linear regression (y=−0.01416*x + 0.3287) were significant (F_(df=1.6)_ = 35.17, p < 0.001, Fig. 2A). After normalization for body mass change (Fig. 2B). its linear regression (y=−0.01416*x + 0.3287) was no longer significant (F_(df=1.4)_ = 0.537, p = 0.504).

**Figure 3:**
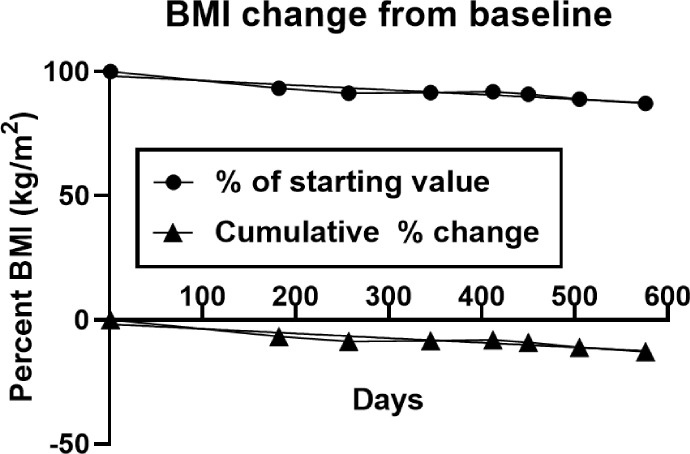
Absolute (solid circles) and relative (solid triangles) changes in the BMI from the baseline.

BMI change is shown in Fig. 3. The absolute BMI decline over 82 weeks was 3.3 kg/m^2^ or 0.04 kg/m^2^/w. The relative BMI decline was 12.8% or 0.16%/w. Both regressions (y=−0.004769*x + 24.98, and y=−0.01876*x + 98.28) were significant (F_(df=1.6)_ = 38.98,p = 0.0008, and F(df=1.6) = 39.47,p = 0.0008).

### Blood parameter changes.

Five of six parameters measured in, or derived from, plasma showed beneficial changes (Table 3). By week 82, fasting plasma glucose declined to 97 mg/dl, HbA1c to 5.6%, LDL-cholesterol to 69 mg/dl (0.6%/w), and triglycerides to 42 mg/dl (0.8%/w). There was no change in HDL-cholesterol (53 mg/dl).

### Appetite changes.

**Figure 4:**
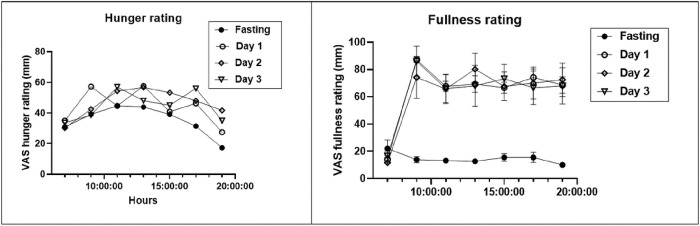
Changes in the VAS ratings of hunger (A) and fullness (B) measured at 2 hour intervals between 7 and 19 hours.

The VAS estimates of hunger and fullness were measured between weeks 32 and 36 at 2-hour intervals during the fasting day and three post-fast days (Fig. 4). For VAS hunger ratings, two-way ANOVA revealed significant time of day differences (p < 0.0001) with lowest assessments at 7 and 19 hours, and overall treatment differences with lowest ratings recorded during the fasting day (p = 0.0036), but no interaction between the time and dietary treatment groups. For the VAS fullness estimates, there was a significant difference between the time of day, groups, and the interaction between time and the dietary treatment with all the lowest ratings during the fasting day, and non-fasting ratings being substantially higher than on the fasting day (p < 0.0001).

**Figure 5:**
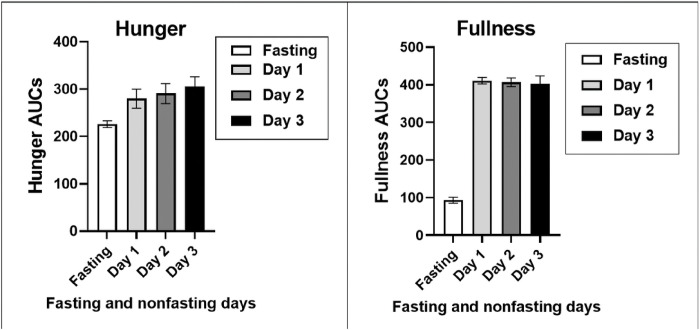
Areas under the curve for hunger (A) and fullness (B) on the fasting day and three subsequent days with ad libitum access to food.

Hunger and fullness AUCs are shown in Fig. 5. Both hunger and fullness AUC ratings differed between the fasting and the three post-fasting days (hunger: F_(df=3,30)_ = 3.8, p = 0.0188, fullness: F_(df=3,30)_ = 246.5, p < 0.0001). Hunger rating AUCs during the fasting day were 19.3, 22.1, and 26.1% lower than on the post-fast ad-libitum eating days, D1 through D3. Fullness ratings were 77.3, 77.1, and 76.8 lower during the fasting day than on the three post-fast days (Fig. 5).

### Blood pressure.

**Figure 6:**
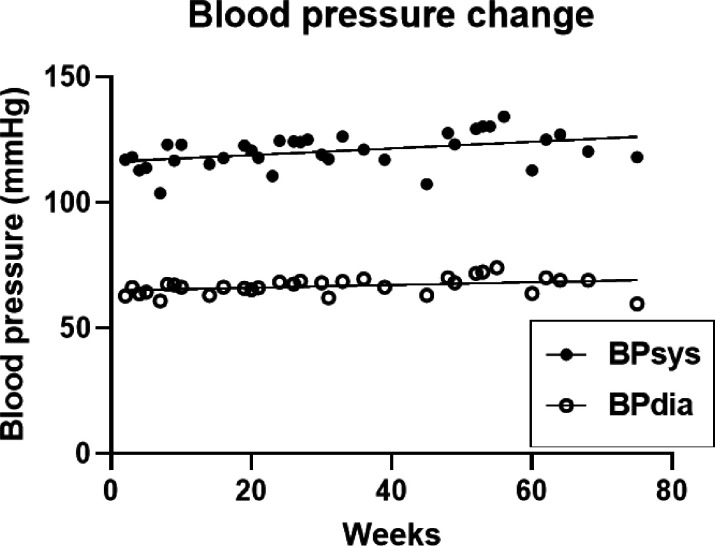
Time course of changes in systolic and diastolic BP measurements. BP dia = diastolic blood pressure; BP sys = systolic blood pressure; Hg= mercury.

The average systolic (BPsys) and diastolic (BPdia) blood pressure during 76 weeks of measurements was 125.5 ± 1.18 and 66.7 ± 0.6 mmHg, respectively. Linear regression analysis revealed that both the BPsys (F_(df=1,33)_ = 7.085, p = 0.0119) and BPdia (F_(df=1,33)_ = 4.375, p = 0.045) significantly increased (Fig. 6). The total 76-week increases for BPsys and BPdia were 10 and 4 mm Hg, respectively.

### Changes in the measures of energy expenditure.

**Figure 7:**
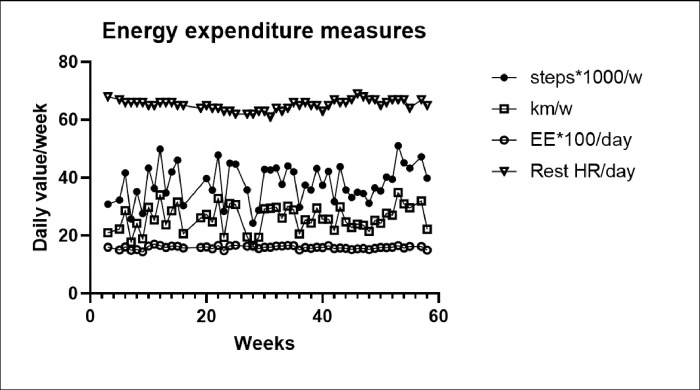
energy expenditure values. EE=energy expenditure; Rest HR/day= resting heart rate per day.

None of the five measures of energy expenditure changed significantly over 82 weeks of the study (Fig. 7). Mean weekly measures for steps (35,873.6 ± 742.8) and kilometers walked (24.4 ± 0.5), total energy expended (1587.3 ± 6.5), and resting heart beats during sleep (64.9 ± 0.2) changed over 82 weeks by a total of only 2.8 steps, 1.1 km, 0 kilocalories, and 0.02 heart beats, respectively. None of their respective linear regressions (steps: y = 4.974*x + 36931, kilometers: 0.01357*x + 25.13, kilocalories:−0.1626*x + 1597, and heartbeats:−0.0002101*x + 65.18) were significant (F_(df=1,70)_ = 0.02211, 0.419. 0.3126, and 0.0005318).

**Figure 8:**
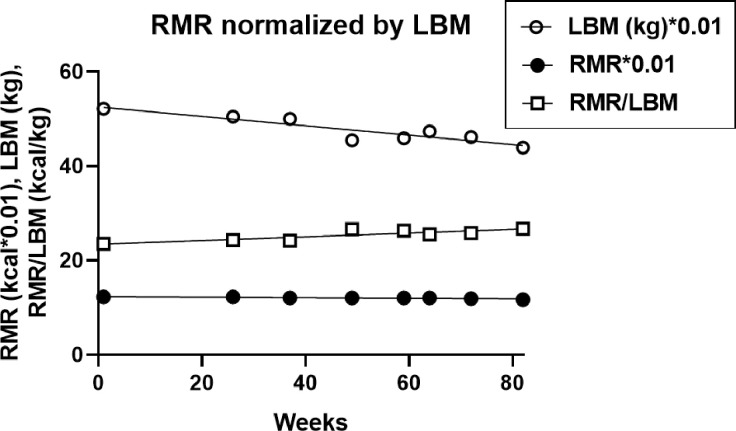
Changes in RMR, LBM and their ratio. RMR= resting metabolic rate, LBM= lean body mass.

Mean 82 week resting metabolic rate was 1,206.3 ± 6.1 kilocalories and declined by only 6.5 kcls over that period. Although absolute LBM decline was significant, its decline relative to changes in total body mass was not. As a consequence, RMR change normalized by LBM of 25.4 + 0.5 kcal/kg) was a non-significant 0.3 kcal/kg increase over 82 weeks (Fig. 8).

### Sleep changes.

**Figure 9:**
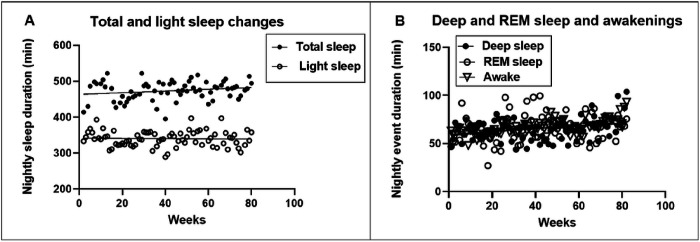
Total nightly minutes sleep of total and light sleep (A), and of deep and REM sleep and minutes of wakefulness during the sleep period (B).

Different measures of sleep are shown in Figs. 9A (total and light sleep) and 8B (deep and REM sleep and duration of wakeful time during the sleep period). Mean durations of total, light, were 473.8 ± 3.1 minutes or 7.9 hours, 340 ± 2.5 minutes or 5.7 hours, and for deep and REM sleep, and wakefulness during sleep period, respectively 64.6 ± 1,4, 66.7 ± 1.7, and 66 ± 0.9 minutes or 1.1 hours. The percentages of light, deep, REM sleep, and of the period of wakefulness as a function of total sleep duration were 71.8, 13.6, 14.1, and 13.9, respectively. Mean number of brief awakening episodes during sleep period was 4.5 ± 0.1, or one percent of total minutes of sleep. The slopes of linear regressions changed significantly during 82 weeks of measurements only for deep sleep (F_(1,76)_ = 4.205, p = 0.0437), wakeful periods (F_(1,76)_ = 37.08, p < 0.0001), and episodes of brief awakenings ( F_(1,76)_ = 32.29, p < 0.0001, Fig. 9B) but not for the total (F_(1,76)_ = 3.052, p = 0.0851), light (F_(1,76)_ = 3.052, p = 0.794), or REM sleep (F(_1,76)_ = 2.489, p = 0.1189). Over 82 weeks, total sleep and light sleep decreased by a total of 19.2 and 2.3 minutes, and deep and REM sleep and duration of wakefulness increased by 8.6, 8.9, and 15.9 minutes, repectively. Nightly awakenings increased by 3 minutes. Average sleep efficiency, estimated as hours of wakefulness divided by hours of sleep [32] was 13.9%, with an increase of 6.2 efficiency units in 82 weeks.

### tBeta-hydroxybutyrate (BHB) changes.

**Figure 10:**
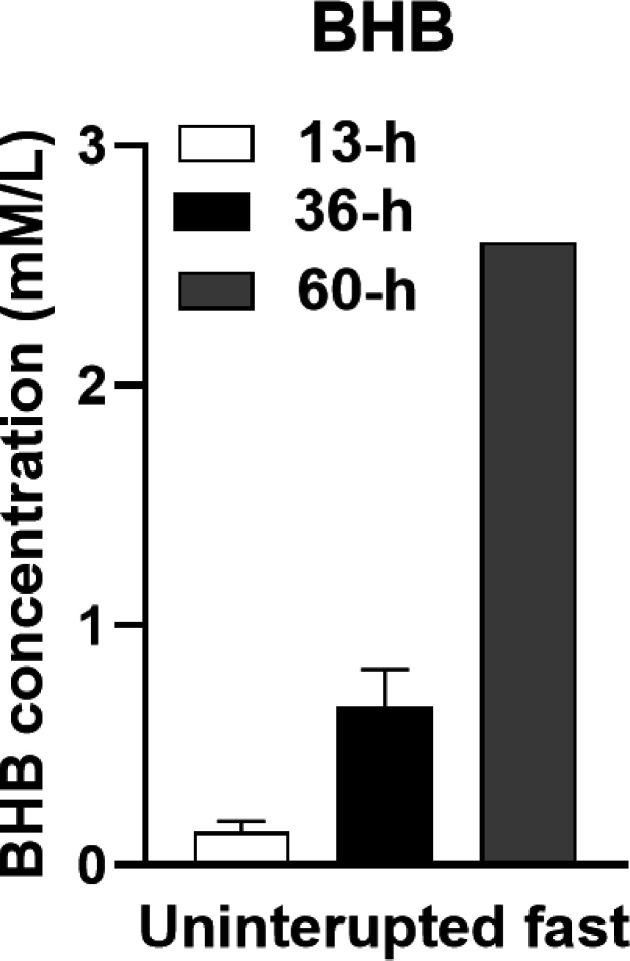
BHB concentrations as a function of 13, 35, and 60 hours of uninterrupted fasts. BHB= beta hydroxybutyrate, a ketone body; H=hour; L= liter; mM= millimoles

Figure 10 shows mean BHB concentration on the morning after a 12-h overnight UF, after 36 hours of near-absolute fast on non-consecutive days, and after 60 hours of near absolute fast on two consecutive days.

As shown in Fig. 10, BHB concentration after 36 hours of near-absolute fast was 0.66 ± 0.07 mM/L, 4.4 times higher than the 0.14 ± 0.02 mM/L measured after the 12-hour overnight fast on non-fasting days. A 60-h UF following two consecutive days of eating fewer than 100 kcal, produced a BHB concentration of 2.6 mM/L, 17.3 times higher than on free-feeding days.

## 3. Discussion of the 5:2-NC IF protocol results

Eighty-two weeks of near-absolute 36-hour fasts on two nonconsecutive days per week revealed substantial changes in several anthropometric, blood pressure, and BHB measurements, variable changes for components of appetite and sleep, and no change in the measures of energy expenditure.

### Anthropometric measures.

Weight loss was sustained over 82 weeks at an average rate of −0.1kg/w (Table 3), but it was not linear (Fig. 1B). During the first ten weeks it was at its fastest (−0.25 kg/w), but there were periods of slower loss, weight stabilization, and even weight gain. The time course of fat mass loss was more consistent, also at −0.1 kg/w (Fig. 2A), but it was no longer significance was lost after normalization by body mass (Fig. 2B). LBM also declined at −0.1 kg/w, but its significance when normalized by whole body mass, (Fig. 2B). The rate of decline in BMI was 0.04 kg/m^2^ or 0.16% per week (Fig. 3). All measures of plasma chemistry displayed various degrees of improvement.

### Appetite.

There is considerable interest in the extent to which IF influences appetite. A number of studies reported increased hunger after prolonged fasts [4–9]. Increased post-fast hunger is considered one of the components of MDM driving increases in food intake which obstruct weight loss and promote weight regain. VAS measurements, carried out between weeks 32 and 36 of weight loss, the period of lowest midpoint weight level, revealed a small decline in hunger ratings (p = 0.004) on fasting days relative to non-fasting days, and a very strong and consistent suppression of fullness ratings (p < 0.0001) (Fig. 4). This contrast was also evident in the comparison of fullness and hunger AUCs (Fig. 5) where hunger AUCs were only 19 to 26% lower on fasting days than on feeding days (F = 3.8, p = 0.02), while fullness AUCs were 77% lower (F = 246.5, p < 0.0001). These data suggest first, that declines in fullness are greater and better detected than in those in hunger, and second, that the 5:2-NC IF protocol characterized by 36 hours of near absolute fast twice a week does not produce a significant increase in hunger after a 5.2-kg body weight loss.

### Blood pressure.

Significant increases in systolic and diastolic blood pressure over 82 weeks of 5:2 IF were unexpected based on their reported negative correlation with body fat [33]. However the 82-week mean BP value was a healthy 120.5 ± 1.2 systolic and 66.7 ± 0.6 mm Hg diastolic.

### Energy expenditure.

Measures of energy expenditure over 82 weeks of 5:2-NC IF were consistently stable. No significant changes were detected in either number of weekly steps, kilometers walked, daily energy expenditure, or resting heart rate. RMR declined by only 6.5 kcal over 82 weeks, an effect that disappeared after normalization for LBM change. It therefore appears that sustained slow weight loss using this IF protocol produces losses in body fat, LBM, and RMR that are proportional to losses in total body mass while the measures of energy expenditure remain stable. Our data are, therefore, supportive of the constrained model of energy regulation which posits that energy expenditure is regulated and protected at the expense of body mass, fertility, growth, and immune and stress defenses as reported by others.[16, 17]. Our data also do not support the hypothesis that this IF protocol suppresses the operation of MDM as suggested by great variability in the rate of weight loss that was at times suppressed or even reversed.

### Sleep variables.

Sleep parameters were stable and within the healthy absolute ranges. Although the slopes of deep sleep, wakefulness during sleep period, and number of brief awakenings linear regressions, were significant, clinical relevance of these changes to overall health is questionable because of the small magnitude of absolute changes (8.6, 15.9, and 3 minute increases over 82 weeks, respectively).

### BHB concentrations.

A very clear and significant change was a 4.4-fold rise in BHB concentration in the mornings after 36-hours of near absolute fast compared to value achieved after a 12-h fasts. Given that BHB concentration asymptotes at 7 mM/L after 25 hours of absolute fasting [25, 26], the rise in BHB concentration from 0.14 mM/L after a 12-hour fast to 0.66 mM/L after a 36-hour fast, and to 2.6 mM after a 60-hour fast clearly shows a strong association between the concentration of BHB and the duration of near-absolute UF (Fig. 10).

### Conclusion.

Self-directed twice weekly near-absolute 36-hour fast can be conducted relatively comfortably and effectively over an extended period of one and a half year. It produces a sustained, but modest and variable rate of body mass loss, a slight decrease in hunger with a greater decrease in fullness, modest increases in blood pressure, no changes in measures of energy expenditure or sleep phases, and 4.4-fold increase in the concentration of BHB.

## 4. Materials and methods for the M4:3-NC protocol.

Of the ten modified IF studies thatimplemented fasting on three nonconsecutive days per week, four engaged independent groups of subjects [34–36, 40] which provided information on weight and fat loss and other physiological and metabolic changes. Three [35–36, 40] matched the dietary restriction in the IF and DCR groups, and the fourth [34] did not employ a non-IF control group. The other 6 studies [37–39, 41–43] were secondary analyses of additional effects of this IF protocol collected from the subjects examined in study [36]. (Table 2)

The distinguishing feature of M4:3-NC studies was that they all offered a 500 to 600 kcal meal in the morning of the three NC fasting days per week. Overall weekly caloric restriction was 30 to 33% in the four IF protocols and, with the exception of study [34], in their DCR controls. As was the case in 5:2 -NC IF protocol, M4:3-NC protocol generated a F/E ratio of 1.8 derived from 108 hours of fasting and 60 hours of eating per week. The two isocaloric IF protocols however differed in the duration of UF. The full-size 500–600 kcal meal interrupted the previous 12 hours of nocturnal fast, reducing the UF to 20 hours in M4:3-NC on fasting days (8 h during fasting days and 12 hours during subsequent nocturnal fast) and 12 h of UF in isocaloric DCR controls. Baseline characteristics of the subjects and variables common to both 5:2-NC and M4:3-NC protocols are shown in Table 2. Participant numbers ranged from 14 to 25 per group. They were between 33 and 51 years old (40.8 ± 4.9), and all were overweight to obese (mean BMI of 33 ± 0.9 kg/m^2^). Study duration varied between 6 weeks [34], eight weeks [36–39, 41–43], and 12 weeks [35 and 40], of which study [40] also had a 12-week follow-up period with no imposed fasting.

The principal aims of the ten studies differed and to a variable degree shared the outcomes measured in 5:2-NC protocol. Four of the ten M4:3-NC studies [34–36, 40] measured weight and fat loss which was the principal aim in studies [34] and [40]. The remaining 6 studies [37–39, 41–43] were secondary analyses of different variables but based on the subjects in study [36]. Variables not shared with the 4:3-NC protocol included insulin sensitivity and metabolic risk factors [36], tissue inflammation [37], circadian clock genes in muscle and adipose tissue [38], markers of lipid metabolism in skeletal muscle [39], mood, cognitive function, and quality of life [41], markers of autophagy in the muscle of humans and muscle and liver of mice [42], and growth differentiation factor 15 [43 Only data common to 5:2-NC and M4:3-NC was included in the protocol comparisons.

Variables used in comparisons between protocols 5:2-NC and M4:3-NC were (a) body weight and body fat losses reported in studies [34–36, 40]; (b) parameters of energy expenditure in study [35] such as changes in RMR, efficiency of energy utilization by muscle (EEf), and lipid utilization through measurement of respiratory quotient (RQ, ration of C)2 produced to oxygen consumed); (c), concentrations of BHB in [36]; (d) blood pressure changes in [34]; (e) appetite ratings evaluated by Three-factor eating questionnaire (44) and reports about the levels of assigned and actual food intake [36, 43]; and (f) quality of sleep assessed by Pittsburgh Sleep Quality Index (45) in study [41].

## 5. Comparison of results between the M4:3-NC IF and DCR protocols

Table 3 presents results of the ten M4:3-NC IF protocols and their DCR controls in columns 2 through 5. IF70 anthropometric data from the 6 secondary analyses [37–39, 41–43] based on data collected from subjects in study [36] are shown in column 2 (referred to as Seven studies), and data from their DCR controls in column 3. They were separated from data from subjects in studies [34, 35, and 40] (referred to as Three studies) because the IF70 group in study [36] consumed 188 fewer calories per day than expected [33, 43] rendering the caloric restriction in Seven studies greater (37%) than in their DCR controls (32%). In the remaining Three studies [34, 35, and 40], the degree of caloric restriction in the IF70 and DCR70 controls was matched, and their results are shown in columns 4 and 5, respectively.

The mean starting body mass, fat mass, percent body fat, and LBM of IF and control DCR subjects in the Seven studies were 89 ± 0.1, 43 ± 0.2, 48.7 ± 0.3, and 51.1 ± 6.4, respectively. In the Three studies, the respective starting values of IF and control subjects were: 92 ± 5.4, 43.5 ± 1.9, 48.9 ± 2.1, and 53.5 ± 4. Starting BMI values in the Seven and Three studies were 33.3 ± 0.9 and 30.2 ± 0.8, respectively. With the exception of blood pressure determinations measured in two studies [34, 40], the remaining variables, including hunger, circulating metabolites and BHB concentration [34], efficiency of energy utilization [33], and parameters of the appetite and sleep [41] were documented in single studies. The starting values for these variables were: BPsys: 118.9 ± 2.4, BPdia: 75.6 ± 3.8 mm Hg, f glucose: 88.2 ± 0 mg/dl. LDL-cholesterol: 114.1 ± 2, HDL-cholesterol: 54.1 ± 0, plasma TG: 110.7 ± 4.4 mg/dl. No baseline assessments of BHB concentration, appetite measures of dietary restraint, disinhibition, and hunger using a Three-factor eating questionnaire [44] or of sleep quality using Pittsburgh Sleep Quality Index [45] were provided.

Table 3 displays the weekly rate of changes in measured variables. Mean weekly losses in body mass, fat mass, and LBM in all studies ranged between 0.5 and 0.9, 0.4 and 0.8, and 0.1 and 0.2 kg, respectively, but differed between the Seven IF and the Three IF studies. Comparing first the groups that underwent 30 to 35% weekly energy restriction (DCR subjects in Seven studies and both IF and DCR subjects in Three studies), weekly body mass and body fat losses were approximately equal between DCR and IF groups (11.1 to 12.5% difference). The more severely restricted IF70 subjects in Seven studies lost 37.5 to 44.4% more of body mass per week, and 40 to 45% more of fat mass/w than their less energy deprived controls. BMI loss was similar in the IF70 and DCR70 subjects in the Seven studies, but 25% greater in the DCR70 subjects in the Three studies. Among the blood metabolites, measured only in subjects in [36], final concentrations of fasting glucose, LDL cholesterol, HDL cholesterol, and plasma triglycerides for the IF subjects were 83.2, 97.8, 50.3, and 86.8 mg/dl, and for their DCR controls 88.2, 111.4, 51.8,and 104.5 respectively. Thus, mean weekly declines in fasting glucose in IF subjects were 0.6 mg/dl/w or 0.75%/w, and in their DCR70 subjects, 0.01 mg/w or 0.01%/w. For plasma lipids, corresponding declines in the two groups were, respectively, 2 mg/dl or 1.8%, and 0.3 mg/dl or 0.3% for LDL cholesterol, 0.5 mg/dl or 0.9% and 0.3 mg/dl or 0.6% for HDL-cholesterol, and 3 mg/dl or 3% and 0.8 mg/dl or 0.7% for plasma triglycerides. A single BHB measurement [36] after 8 weeks of IF was 0.03 mM/L after a 12 h fast and 0.26 mM/L after a 20-h fast. In DCR subjects, the morning BHB value was 0.02 mM/L. RMR, RQ, and energy efficiency were measured in [35] subjects within the group of Three. Although the weekly decline in the RMR in the IF70 subjects was about threefold greater than in their DCR70 controls, but after normalization by the LBM, RMRLBM substantially diminished or became positive. Increased utilization of lipids for energy, as measured by RQ, was equal in the IF70 and DCR70 groups. Net energy efficiency of muscle energy utilization during increases in mechanical resistance while cycling at 10, 25, and 50W rose significantly in the IF70 subjects (y = 0.0053*x + 0.0113), but not in the DCR70 controls (y = 0*x + 0.0063). Finally, there was a small non-significant increase in TFEQ score (1.1) in the IF subjects, but not in the DCR70 subjects (0), and no change in the quality-of-sleep scores in the single Seven-group study [41].

### Conclusion.

Where the degree of caloric restriction was equivalent in IF70 and DCR70 groups [34, 35, and 40], the weekly rate of weight, fat, and the BMI losses were similar between the groups. Where the IF70 subjects were more severely food restricted [36–39 & 41–43], weekly losses in these variables were greater in the IF than in the DCR70 subjects. Plasma metabolites were measured only in study [36]. Declines in plasma triglycerides were 3.75 times greater, and in LDL cholesterol almost 6 times greater, in the IF subjects in the more severly restricted Seven-study subjects than in their DCR controls. In one of the Three isocaloric restriction studies [35], RMR was three times greater in IF70 than in the DCR70 group, but substantially declined or became positive after normalization by LBM. In studies [34] and [40], declines in systolic and diastolic blood pressure were of approximately equal magnitude in IL70 subjects but about 1.5 to 1.6 times greater than in DCR70 subjects. Net energy efficiency increased during stepwise increase in muscle loading during cycle ergometry in the IF, but not in the DCR group. No group differences were seen in TFEQ appetite scores and PSQI sleep scores were not affected even in more severely energy deprived subjects.

## 6. Discussion of the outcome differences between the M4:3-NC and 5:2-NC IF protocols

Before discussing the outcome differences between the ten M4:3-NC studies and a single 5:2-NC case report, it is necessary to point out the limitations of such a comparison. The limitations are many. Starting with study subjects, there were significant differences: in age almost by a factor of 2 (44.6 ± 2.6 vs 82 years), in starting body mass: 88.7 ± 1.1 vs 70.9 kg, in fat mass: 42.2 ± 0.5 kg vs 20.9 kg, in percent body fat: 45.0 ± .0 vs 32.8, and in the duration of IF intervention: between 6 and 12 weeks vs 82 weeks. Other significant limitations are that, with the exception of body mass and body fat changes, most variables in M4:3-NC, eligible for comparisons to protocol 5:2-NC, were analyzed in very few or single studies. Additional limitation is that Seven M4:3-NC studies [36–39, 41–43] were based on a single group of IF subjects measured in study [36], and that their IF group was more severely energy deprived (37%) than their DCR controls (32%). Only in Three studies [34, 35, and 40] was energy restriction in IF70 and DCR70 controls matched. Despite these limitations, the usefulness of the outcome comparisons is in revealing a pattern, and suggesting the direction, and magnitude of IF-induced changes as a function of a difference in the duration of UF in studies matched for F/E ratio.

The first hypothesis that, due to equal F/E ratios, 5:2-NC, M4:3-NC protocols, and isocaloric DCR controls will exhibit comparable rates of change in anthropometric variables including weight, fat, and LBM losses, was not supported. The weight loss studies are predicated on the assumption that the magnitude of weight loss will bear a linear relationship to the quantity of restricted calories without the interference of an MDM. In the present study, the M4:3-NC (columns 2 through 5 in Table 3) and 5:2-NC protocol (column 6) were matched for weekly F/E ratio of 1.8 (60 hours of feeding to 108 hours of fasting per week) with the expectation of an equal rate of weight loss. In contrast to the expectation, weekly rates of body mass loss in the M4:3-NC protocol was about seven to nine times higher (0.7 to 0.9 kg/w) than in the 5:2-NC protocol (0.1 kg/w). The weekly rate of weight loss in the Three isocalorically-matched IF70 and their DC70 controls was approximately equal (0.9 and 0.8 kg/w, respectively).The near equality between the IF70 and DCR70 rates of weight losses in the three well-matched groups [34, 35, 40] confirms the conclusion reached in a systematic review of IF studies [46] that intermittent fasting appears to produce similar effects to continuous energy restriction (DCR) in reduction of body weight. One possible explanations of the difference in the rate of the weight losses in M4:3-NC and 5:2-NC protocols may have to do with differences in the duration of the comparison studies. The ten studies in the M4:3-NC protocol were between 6 and 12 weeks long (with only 2 studies lasting 12 weeks), while 5:2 protocol extended over 82 weeks. As Fig. 1B shows, the rate of weight loss was faster at −0.28 kg/w during the first 10 weeks of IF in 5:2-NC study than during weeks 10 to 60, when it averaged 0.04 kg/w and included periods of no loss or even some weight gain. This prompts a speculation that higher rates of weight loss in M4:3-NC than in 5:2-NC protocols are attributable to the faster rates of body mass losses during the early stage of fasting.

The second possible explanation for the difference in the rate of weight loss between the two protocols, may be related to their difference in the starting body fat mass. Like the changes in the rate of body mass loss, fat loss also was 5 to 8 times higher (0.5 to 0.8 kg/w) in the M4:3-NC subjects than in those in 5:2-NC protocol (0.1 kg/w). Body fat measurements in 5:2-NC study were less frequent, but the rate of fat loss during the first 26 weeks of IF was faster at 0.3 kg/w than during the next 38 weeks at 0.06 kg/w. Most of the M4:3-NC studies did not display continuous weight or fat measurements, so it can be speculated that at least some of the difference in the fat-loss rates between the two protocols are attributable to the faster rates of losses during the early stages of fasting. In Seven studies, fat losses were 25% greater in the more heavily food-restricted IL subjects than in their DCR-70 controls. In the Three studies fat losses were about equal in the isocaloric IF70 and DCR70 subjects, again confirming the conclusion from a review of IF studies [46] that intermittent fasting appears to produce similar effects to DCR in reduction of body fat. However, while there is ample evidence that the magnitude of energy restriction affects the rates of weight and fat losses ([3] and columns 2 vs 3 in Table 3), there is a possibility, which is not much explored, that the size of the original body and fat masses affects the rate of body mass and body fat losses. The starting differences in these body compartments were 89.1 ± 0.1 for body mass and 43.3 ± 0.2 kg for body fat (48.7%) in the M4:3-NC studies and 70.9 and 29.9 kg (32.8% fat), respectively, in the 5:2 case report.

Most weight loss strategies aim for the losses of body weight and body fat, but for the preservation of LBM. It is of interest that losses of the LBM were about equal not only between IF70 and DCR subjects in both group-of-Seven and group-of-Three studies, but also in the 5:2-NC protocol. It is also of interest that the magnitude of LBM loss in the 5:2-NC study was of the same magnitude as the rates of weight and fat loss in this protocol, while in the M4:3-NC protocol the losses of body mass and body fat were between 7 to 9 times greater than the losses of LBM. This would support the speculation that the size of the starting weight and fat masses influences the rates of their loss, but not the rate of LBM loss.

Two sets of changes that usually accompany weight and fat loss entail changes in circulating metabolites and blood pressure. The starting values in these variables showed some differences between subjects in the two protocols. The fasting glucose was 11 percent lower, and LDL cholesterol 13% lower, in M4:3-NC than in 5:2-NC subjects, while plasma triglycerides were 1.7 times higher in this protocol than in the 5:2-NC study. In both protocols, all of the blood metabolite concentrations declined except for the largely unchanged HDL-cholesterol. In the M4:3-NC. The changes were more pronounced in the IF subjects than in their less food restricted DCR70 controls. In the IF subjects, fasting glucose changed from 88.2 to 83.2 mg/dl (0.75%/w), and LDL-c, HDL-c, and TGs changed from 114.1 to 97.8 (1.8%/w), 54.1 to 50.3 (0.9%/w), and 110.7 to 86.8 mg/dl (21.6%/w), respectively. In the DCR controls, these changes were 88.2 to 88.1 for fasting glucose (0.1%), 114.1 to 111.4 (0.3%/w), 54.1 to 51.3 (0.6%/w), and 110.7 to 104.5 mg/dl (0.7%/w), respectively. In the 5:2-NC protocol, fglucose changed from 99 to 97 (0.02%/w) and HbA1c from 5.8 to 5.6. LDL-c changed from 131 to 107 (0.2%/w), HDL-c remained at 53, and TG changed from 66 to 42 mg/dl (0.4%/w). However the changes were of little clinical significance because they all remained below the unhealthy cutoff values of 100 mg/dl for fasting glucose, and 130 and 150 mg/dl for LDL-cholesterol and plasma triglycerides, respectively, in both protocols.

Starting systolic blood pressure was comparable in the two protocols at about 116 mm Hg, but the diastolic pressure was 13% higher in M4:3-NC subjects than in the 5:2-NC subject. The divergence in the BP changes in the two protocols, with both pressures decreasing in the M4:3-NC subjects, and increasing in the 5:2-NC study, may be attributable both to the difference in starting level of body fatness (BMIs of 25.4 vs 32.8 ± 0.6 kg/m^2^), respectively, and in subject age (44.6 ± 2.6 vs 82.5 years). Blood pressure change was reported to be positively related to body weight and fat losses [33], while advancing age has been negatively related to blood pressure, more so in women compared to men [47]. In both protocols, blood pressure values before and after the IF exposure remained largely within the healthy clinical range, where the ideal systolic to diastolic BP is represented by 90/60 mm Hg, and normal healthy range by 140/90 mm Hg.

Additional variable, often linked to changes in anthropometric values, is quality of sleep. No changes were observed in the quality of sleep in the M4:3-NC study [41]. A more detailed analysis of sleep patterns in the 5:2-NC protocol uncovered almost no changes in the duration of total (473.8 min), light (340 min), and REM sleep (64.6 min) with only − 19.2, −2.3, and 8.6 min over 82 weeks. Increases of 8.6, 15.9, and 3 minutes respectively for deep sleep (66.7 min), periods of wakefulness (66 min), and number of awakenings (4.5) over 82 weeks were statistically significant albeit very small.

Second hypothesis posited that the longer 36-h duration of UF in the 5:2- NC IF protocol will affect manifestations of MDM including increased hunger and efficiency of muscle EE, and decreased RMR and levels of voluntary activity. Data addressing this hypothesis were provided by one study in the group of Three [35] and several measurements in the protocol 5:2-NC. The [35] study defines MDM as a reduction in total energy expenditure, driven by both a decline in RMR, non-resting EE, reduced spontaneous physical activity, and an increase in exercise energy efficiency. Additional contributors to MDM are increased hunger and drive to eat, and reduced fullness and fat oxidation.

Hunger was briefly characterized in study [36] as being lower on fed day in IF70 than in DCR70 subjects, but there was no mention of the sensation of fullness. A composite score reflecting dietary restraint, disinhibition and hunger components, but no clear assessments of hunger and fullness, from a questionnaire [41] reported no significant difference between IF (1.1) and DCR70 (0) responses. By contrast, appetite was fully characterized in 5:2-NC case report as producing slightly, but still significantly lower, VAS hunger ratings (p = 0.004), and a very strong and consistent suppression of VAS fullness ratings (p < 0.0001) on fasting days. On fasting days, AUCs of hunger ratings were 19 to 26% lower than on feeding days (F = 3.8, p = 0.02), while fullness AUCs were 77% lower (F = 246.5, p < 0.0001). A systematic review and meta-analysis of the effect of IF on appetite [48] concluded that there was no clear evidence that this fasting protocol affected hunger. Thus, both the negative evidence regarding changes in hunger in the protocol 5:2-NC and in study [41] and conclusions in the review [48] do not justify modifying the weekly IF protocols by addition of the 500 to 600 kcal meals on fasting days with the expectation of increased hunger.

Addition of a 500 to600 kcal meal of fasting days in modified weekly IF protocols is not only unnecessary, but also detrimental in that it curtails increases in BHB which rise in proportion to the duration of UF. In the study [34) supporting only 20 hours of UF because of the large meal on fasting days, the increase in BHB was only to 0.26 mM/L in the morning after the fasting day. In protocol 8:2-NC which allowed an UF of 36 hours of near absolute fast, the increase in BHB at 0.66 mM/L was 2.5times greater. In view of the powerful signaling functions [28] of BHB, it would be beneficial to explore the health benefits of weekly fasting IF protocols with longer UF periods. BHB was shown to epigenetically activate gene networks that turn on lipid metabolism, enzymes that promote energy mobilization and utilization, and mitochondrial biogenesis, while suppressing biosynthetic pathways, and eliciting sirtuin 1 release [29],

Total EE and RMR and spontaneous physical activity were measured in 5:2-NC protocol, and RMR, lipid oxidation, and exercise energy efficiency in study [35]. Both kilocalories of total EE and of RMR in 5:2-NC study, and of RMR in [35] study were threefold greater in absolute terms than after normalization by LBM, when the changes became negligible or even positive. Remarkably, net efficiency of energy utilization (NE) during increases in mechanical resistance while doing bicycle ergometry rose significantly in the IF70 subjects as they cycled at 10, 25, and 50W (y = 0.0053*x + 0.0113), but not in the DCR70 controls (y = 0*x + 0.0063). However, increased exercise efficiency was previously reported [13] in subjects who were maintained at a10 percent lower than normal weight achieved by the DCR method. The difference between the two studies cannot be explained as resulting from different magnitude of weight loss, because it was greater in [35] at 15% in IF group and 14% in DCR group, but may have possibly been caused by the difference in the extended period during which the 10% body weight was maintained in study [13]. There was no effect of IF on lipid utilization as measured by RQ [35].

Protocol 8:2-NC provided evidence that there were no differences in the levels of voluntary physical activity. The number of weekly steps and kilometers walked remained relatively constant over 82 weeks. Adding this to the evidence that total EE, RMR remain constant in both protocols supports the concept that energy expenditure is regulated in conjunction with the maintenance of LBM despite the losses of body mass and body fat. This hypothesis posits [16, 17], that to preserves total energy expenditure during energy restriction, MDM operates by making muscle contractions more efficient (13) and by suppressing energy-costly processes supporting immunity, reproduction, growth, and stress responses (16,17). We find no evidence that IF reduces or mitigates this process.

## 7. Conclusions

The 5:2-NC protocol, that allows 36 hours of near absolute UF twice a week, supported slower, but sustained and variable rates of weight and fat losses, slightly lower sensation of hunger but a substantial decline in fullness, and about 2.5-fold higher post-fast concentration of the ketone body BHB than the isocaloric modified three-days a week fasting protocol, M4:3-NC, that allowed 20 hours of UF. The 5:2-NS study slightly reduced hunger and more profoundly reduced fullness. Addition of the large meal to modified weekly IF protocols in expectation of increased hunger, is therefore not necessary and carries a disadvantage of reducing the rise in BHB, an important signaling molecule with a number of epigenetic benefits.. IF protocols did not change the parameters of energy expenditure, or counteracted manifestations of a MDM that interferes with weight loss and promotes weight regain. Self-directed 5:2-NC protocol was comfortably tolerated over 82 weeks.

## Figures and Tables

**Table 1:** Baseline variables.

Variable	
Age (years)	82
Reproductive status	Post menopause
Body mass (kg)	70.9
Height (m)	1.67
BMI (kg/m^2^)	25.4
Fat mass (kg)	29.9
Fat (%)	32.8
Lean body mass (kg)	52.2
Fasting glucose (mg/dl)	99
Hemoglobin A1c (%)	5.8
LDL-cholesterol (mg/dl)	131
HDL-cholesterol (mg/dl)	53
Cholesterol/HDL ratio	2.5
TG (mg/dl)	66
BP systolic (mm Hg)	116.2
BP diastolic (mm Hg)	65.1
RMR (kcal/day)	1,226
Energy expenditure (kcal/day)	1,598
Steps walked /w	35,510
Distance walked (km/w)	24.6
Total sleep (min/night)	414
Light sleep (min/night)	333
REM sleep (min/night)	46.8
Deep sleep (min/night)	46.8
Duration awake (min/night)	60

BMI = body mass index (kg/m^2^); BP = blood pressure; HDL = high-density Hg = mercury; kcal = kilocalorie; kg = kilogram; km = kilometer (1,000 m); LDL = low-density; m = meter; min = minutes; RMR = resting metabolic rate; TG = plasma triglycerides; w = week.

**Table 2:** Baseline characteristics of the ten M4:3-NC studies and the participant subjects.

Study ID	Study	Study duration (FU) w	Subject number (BMI)	Age (years) sex	FR in DCR controls	Comparison data
34	Eshghinia & Mohammadzadeh 2013	6	15 (33.2)	33.5 W	none	BM, BP, FM,
35	Coutinho et al. 2017	12	14ea (35.3)	39	33% DCR	BM, EEf, FM, RMR
36	Hutchison et al. 2019	8	25 ea (32.3)	50 W	30% DCR	Hunger, BHB, BM, fG,FM, HDL-c, LDL-c, TG
37	Liu et al. 2019	8	25 ea (32.5)	50 W	30% DCR	BM, FM #
38	Zhao et al. 2020	8	25 ea (32.7)	51 W		BM, FM #,
39	Liu et al. 2021	8	25 ea (32.5)	50 W	30% DCR	BM, FM, BHB, RQ, #
40	Steger et al. 2021	12 (12 FU)	18 IF-17 C (31.2)	NS	DCR	BM, BP FM
41	Teong et al. 2021	8	23 ea (32.9)	50 W	30% DCR	Appetite, BM, FM sleep, #
42	Chaudhary et al, 2022	8	25 ea (ND)	51 W	30% DCR	BM, FM #
43	Liu et al. 2023	8	21 IF (32.6), 24 C (33)	50 W	30% DCR	BM, FM #

BM = body mass (kg); BMI = body mass index (kg/m^2^); BP = blood pressure, DCR or study as a whole; DCR = daily caloric restriction; ea = each group; EE = energy expenditure measures, EEf = efficiency of muscle energy utilization, fG = fasting glucose; FM = fat mass (kg); FR = food restriction; FU = follow-up without IF; IF70 = intermittent fasting resulting in 30% dietary restriction; LDL-c = LDL cholesterol; ND = no data; RQ = ratio of CO2 produced to oxygen consumed; TG = plasma triglycerides; w = weeks; W = women; #=secondary analysis of study [36] data

**Table 3:** Comparison of IF results common to 5:2-NC and M4:3-NC (B) protocols

Protocols	M4:3-NC protocol vs DCR protocol				7:2-NC protocol

Unit or % change/week	IF70 in studies [36–39] & [41–43]	DCR70 in [36–39 & 41–43]	IF70 in studies [34, 35 & 40]	DCR in studies [34, 35 & 40]	Case report

Weight loss (kg)	0.7 ± 0.0	0.5 ± 0.0	0.9 ± 0.2	0.8 ± 0.3	0.11

Weight loss (%)	0.8 ± 0.03	0.6 ± 0.03	1.00 ± 0.1	1.1 ± 0.2	0.15

BMI loss (kg/m^2^/w)	0.2	0.2	0.3 ± 0.0	0.4 ± 0.2	0.04

BMI loss (%)	0.2	0.1	0.2 ± 0.0	0.3 ± 0.1	0.16

Fat loss (kg)	0.5 ± 0.1	0.4 ± 0.0	0.8 ± 0.3	0.7 ± 0.1	0.14

Fat loss (%)	1.2 ± 0.13	0.7 ± 0.1	1.7 ± 0.3	1.7 ± 0.2	0.11

LBM change (kg)	−0.2	−0.1	−0.1 ± 0.4	0.2 ± 0.1	−0.1

LBM change (%)	−0.3	−0.1	−0.1 ± 0.3	0.1 ± 0.3	−0.19

F f glucose (mg/dl)	83.16	88.12	ND	ND	97

F Hb A1c (%)	ND	ND	ND	ND	5.6

F LDL-c (mg/dl)	97.8	111.4	ND	ND	69

F HDL-c (mg/dl)	50.3	51.8	ND	ND	53

F TG (mg/dl)	86.8	104.5	ND	ND	42

F BHB fed/fast (mM/L)	0.03/0.26	−0.010	ND	ND	0.14/0.66

EE (kcal)	ND	ND	ND	ND	−4.1
EE_LBM_ (kcal/kg LBM)	ND	ND	ND	ND	0.6
EE (%)	ND	ND	ND	ND	−0.2
EE_LBM_ (%)	ND	ND	ND	ND	0.2
RMR (kcal)	ND	ND	−10	−3.3	−0.6
RMR (%)	ND	ND	−0.06	+ 0.02	−0.05
RMR_LBM_ (kcal)	ND	ND	−0.07	+ 0.008	0.2
RMR_LBM_ (%)	ND	ND	−0.3	+ 0.03	1.21
Fast RQ (V_CO2_/V_O2_)	ND	ND	−0.004	−0.004	ND
Fast RQ (%)	ND	ND	−0.005	−0.005	ND
EEf@ 10 W,25,50 W	ND	ND	+ 0.005	0	ND

Distance (km/w)	ND	ND	ND	ND	−0.0005

Steps (%)	ND	ND	ND	ND	1.2

BPsys (mm Hg)	ND	ND	−1.2 ± 0.5	−0.7	+ 0.1

BPsys (%)	ND	ND	−1 ± 0.4	−0.6	+ 0.1

BPdia (mm Hg)	ND	ND	−0.9 ± 0.5	−0.5	+ 0.05

BPdia (%)	ND	ND	−1 ± 0.7	−0.7	+ 0.08

Hunger VAS (fast vs D1, D2, D3 fed)	ND	ND	ND	ND	35.2 vs 43.3, 46.7, 48.7

Appetite TFEQ score	TFEQ 1.1	TFEQ 0	ND	ND	ND

Hunger VAS (fast as % of D1, D2, D3 fed)	ND	ND	ND	ND	−81.3, −75.4, −72.3

Hunger AUCc (fast vs D1, D2, D3 fed)	ND	ND	ND	ND	226.1 vs 280.1, 290.8, 306.9

Hunger AUC (fast as % of D1, D2, D3 fed)	ND	ND	ND	ND	−80.7, −78, −73.7

Fullness VAS (fast vs D1, D2, D3 fed)	ND	ND	ND	ND	14.7 vs 63.9, 63.1,63.5

Fullness VAS (fast as % of D1, D2, D3 fed)	ND	ND	ND	ND	−22.7, −23.4, −23.2

Fullness AUC (fast vs D1, D2, D3 fed)	ND	ND	ND	ND	93.2, 410.9, 398.4, 402.1

Fullness AUC (% ch vs D1, D2, D3 fed)	ND	ND	ND	ND	−23, −23.3, −23.1

PSQI score (%)	−9.8	−11.5	ND	ND	ND

TotalS/LS/DS (min)	ND	ND	ND	ND	0.15/−0.22/0.18

TotalS/LS/DS (%)	ND	ND	ND	ND	0.03/−0.06/0.29

REM/WS (min)	ND	ND	ND	ND	0.08/0.20

REM/WS (%)	ND	ND	ND	ND	0.12/0.33

AUC = area under the curve; BHB = beta hydroxybutyrate; BPdia = diastolic blood pressure; BPsys = systolic blood pressure; ch = change; D1, D2, D3 = three post-fast days with ad-libitum access to food; DCR70 = intermittent daily caloric restriction of ~ 30%; DS = minutes of deep sleep, EE = kilocalories of daily energy expenditure; EEf = muscle energy efficiency during exercise; f glucose = fasting glucose; F = final value; HbA1c = hemoglobin A1c); HDL-c = high-density lipoprotein cholesterol; IF70 = daily caloric restriction of ~ 30% with intermittent fasting; km = kilometer; LBM = lean body mass; LS = minutes of light sleep, NC = no change; ND = no data; NE = net efficiency of energy utilization by muscle, increased effort; PP = postprandial period, PSQI = Pittsburgh Sleep Quality Index; REM = minutes of rapid-eye movement phase of sleep; RMR = kilocalories of resting metabolic rate; RQ = respiratory quotient (a ratio between the rate of carbon dioxide production over the rate of oxygen consumption); TFEQ = Three-factor eating questionnaire; TG = plasm triglycerides; TotalS = minutes of total sleep; v = decrease, WS = minutes of wakefulness during sleep period.

## Data Availability

Both can be obtained by request from the author.
